# Preliminary Evaluation of a Conversational Agent to Support Self-management of Individuals Living With Posttraumatic Stress Disorder: Interview Study With Clinical Experts

**DOI:** 10.2196/45894

**Published:** 2023-05-29

**Authors:** Hee Jeong Han, Sanjana Mendu, Beth K Jaworski, Jason E Owen, Saeed Abdullah

**Affiliations:** 1 College of Information Sciences and Technology Pennsylvania State University University Park, PA United States; 2 National Center for PTSD VA Palo Alto Health Care System US Department of Veterans Affairs Menlo Park, CA United States

**Keywords:** conversational agent, PTSD, self-management, clinical experts, evaluation, support system, mental health, trauma

## Abstract

**Background:**

Posttraumatic stress disorder (PTSD) is a serious public health concern. However, individuals with PTSD often do not have access to adequate treatment. A conversational agent (CA) can help to bridge the treatment gap by providing interactive and timely interventions at scale. Toward this goal, we have developed PTSDialogue—a CA to support the self-management of individuals living with PTSD. PTSDialogue is designed to be highly interactive (eg, brief questions, ability to specify preferences, and quick turn-taking) and supports social presence to promote user engagement and sustain adherence. It includes a range of support features, including psychoeducation, assessment tools, and several symptom management tools.

**Objective:**

This paper focuses on the preliminary evaluation of PTSDialogue from clinical experts. Given that PTSDialogue focuses on a vulnerable population, it is critical to establish its usability and acceptance with clinical experts before deployment. Expert feedback is also important to ensure user safety and effective risk management in CAs aiming to support individuals living with PTSD.

**Methods:**

We conducted remote, one-on-one, semistructured interviews with clinical experts (N=10) to gather insight into the use of CAs. All participants have completed their doctoral degrees and have prior experience in PTSD care. The web-based PTSDialogue prototype was then shared with the participant so that they could interact with different functionalities and features. We encouraged them to “think aloud” as they interacted with the prototype. Participants also shared their screens throughout the interaction session. A semistructured interview script was also used to gather insights and feedback from the participants. The sample size is consistent with that of prior works. We analyzed interview data using a qualitative interpretivist approach resulting in a bottom-up thematic analysis.

**Results:**

Our data establish the feasibility and acceptance of PTSDialogue, a supportive tool for individuals with PTSD. Most participants agreed that PTSDialogue could be useful for supporting self-management of individuals with PTSD. We have also assessed how features, functionalities, and interactions in PTSDialogue can support different self-management needs and strategies for this population. These data were then used to identify design requirements and guidelines for a CA aiming to support individuals with PTSD. Experts specifically noted the importance of empathetic and tailored CA interactions for effective PTSD self-management. They also suggested steps to ensure safe and engaging interactions with PTSDialogue.

**Conclusions:**

Based on interviews with experts, we have provided design recommendations for future CAs aiming to support vulnerable populations. The study suggests that well-designed CAs have the potential to reshape effective intervention delivery and help address the treatment gap in mental health.

## Introduction

### Background

Posttraumatic stress disorder (PTSD) is a serious mental health condition that can occur in people who have experienced a traumatic event, such as combat, sexual assault, natural disasters, or other life-threatening situations [1,2]. Approximately, 12 million adults in the United States have PTSD in any given year, and 6% of the US population will have PTSD at some point in their lives [1]. However, many individuals living with PTSD do not have access to the necessary resources for treatment [3]. For example, only 33% of veterans diagnosed with PTSD receive adequate treatment [4]. The treatment gap is caused by a number of significant barriers, including cost and a lack of qualified professionals to provide evidence-based treatments to individuals in need [5].

Recent studies have explored eHealth technologies to deliver mental health interventions in a cost-effective and scalable way [6]. Specifically, there has been an increasing interest in using conversational agents (CAs) to provide support for a wide range of mental health issues, including autism spectrum disorders [7], schizophrenia [8], depression [9-11], and anxiety disorders [9,10,12]. CAs emphasize interactivity, which can lead to better user engagement and more effective support over time. However, while prior studies have explored CAs for PTSD assessment [9] and intervention [13], there has not been much work on using CAs to support continuous self-management of individuals with PTSD. Furthermore, there is a lack of understanding regarding how these CAs might be integrated into existing clinical workflows and practices for PTSD. As such, there is a knowledge gap when it comes to designing and deploying effective CAs focusing on PTSD support.

To address this gap, we have developed PTSDialogue—a CA to support the self-management of individuals living with PTSD [14]. PTSDialogue is designed to be highly interactive (eg, brief questions, ability to specify preferences, and quick turn-taking) and supports social presence to promote user engagement and sustain adherence. It includes a range of support features, including psychoeducation, assessment tools, and several symptom management tools.

In this paper, we describe the findings and outcomes from an expert evaluation of PTSDialogue. Given that PTSDialogue focuses on a vulnerable population, it is critical to establish its usability and acceptance with clinical experts before deployment. Prior work has established that experts’ engagement is essential to ensure the quality and efficacy of CAs for psychiatric treatment [15]. Furthermore, expert feedback is important to ensure user safety and effective risk management in CAs aiming to support a vulnerable population [16]. To evaluate PTSDialogue, we have conducted interviews with 10 clinical experts following their interactions with PTSDialogue. Our data establish the feasibility and acceptance of PTSDialogue to support effective self-management for individuals with PTSD. Design factors and interaction features essential for the success of PTSDialogue have also been identified. Based on these findings, we have outlined design guidelines and requirements for future CAs aiming to support vulnerable populations.

### Prior Work

#### eHealth Technologies Focusing on PTSD Support

A number of recent studies have explored eHealth technologies to support the self-management of individuals living with PTSD. Mobile apps can make it easier to get high-quality psychoeducational materials and self-management tools without needing continuous professional support [17]. For example, the Department of Veterans Affairs has developed a mobile phone app called PTSD Coach [18]. It was created to support PTSD self-management and provide tools for assessment, monitoring, psychoeducation, coping strategies, and crisis support. After a short time of use, PTSD Coach successfully lowers symptom severity [19].

However, most eHealth technologies have a critical issue in terms of low engagement and adherence. For example, PTSD Coach is a widely deployed app, which has been downloaded over 957,000 times in 115 countries around the world [20]. However, it has difficulty sustaining user engagements—only 15.2% of users remain active after 3 months and just 5.5% after a year [21]. High engagement and adherence are generally required for successful intervention to improve health outcomes [22]. Current eHealth technologies for PTSD were unable to sustain longitudinal engagement, which can result in unsatisfactory results and reduce their effectiveness. Given that PTSD also requires continuous self-management, such low user adherence is a key limitation of current technologies to support individuals with PTSD.

To better support engagement and adherence to eHealth technologies, Mohr et al [23] have developed the supportive accountability (SA) model. According to the model, human support enhances the effectiveness of engagement and adherence when providing interventions by eHealth technologies. Specifically, human support can lead to perceived accountability, which can subsequently improve engagement and adherence. For example, Possemato et al [24] evaluated intervention adherence between self-managed PTSD Coach and clinician-supported PTSD Coach. They found that clinician-supported PTSD Coach resulted in high adherence.

However, the SA model poses logistical challenges, which limits its scalability. Specifically, the SA model relies on professionals to provide support and sustain engagement. However, there is a lack of trained professionals to provide mental health support, with there being only 1 active psychiatrist for every 8476 people in the United States [25]. Furthermore, the shortage of mental health professionals is likely to worsen in the coming decades. This shortage is even more concerning in light of the rising prevalence of mental illness in recent years (57.8 million people in the United States as of 2021; 22.8% of all US adults [26]). As such, it is critical to identify alternative strategies to provide support and sustain engagement at scale without adding a burden to the clinicians.

#### Conversational Agents for Supporting Self-management

Recent studies have used CAs to support tailored, engaging interactions with users beyond human support. CAs are defined as dialogue systems that provide support for specific tasks [27,28]. CAs can have different forms (eg, embodied or nonembodied). CAs can use a range of communication modalities, including text, voice, and gestures [29].

Recent studies have explored attributes and features of CAs to support social presence that can lead to better user engagement and adherence. For example, CA interactions with empathetic responses show positive associations between user trust and engagement [30]. Kraus et al [31] found that socially engaged CAs lead to better user trust. Anthropomorphism in CA can also affect user trust. CAs that mirror human-like cues can better support the perceived social presence [32,33]. Verhagen et al [34] also found that friendly language style can impact perceived social presence.

Recent studies have used CAs to provide support for a wide range of mental health issues including autism spectrum disorders [7], schizophrenia [8], depression [9-11], and anxiety disorders [9,10,12]. Specifically, CAs can be particularly useful in mental health assessment. Prior work has shown that CAs can lead to more effective self-disclosure [9,12,35]. For example, DeVault et al [9] developed a web-based interviewer to assess behaviors correlated with anxiety, depression, and PTSD. Recent work has also explored CA attributes that can better support self-disclosure. Lee et al [36] reported that reciprocal disclosure from CAs can support deep self-disclosure from users. Cho et al [37] found that the use of backchanneling cues in CA interactions can lead to a perception of active listening, which was associated with more emotional disclosure from users.

Prior work has also used CAs to support coaching for self-management in mental health illnesses. For example, Morie et al [38] designed a CA to support veterans with PTSD. It aimed to provide a virtual environment for healing activities and social interactions. Tielman et al [13] designed a web-based coach to motivate and support individuals living with PTSD during therapy. Fitzpatrick et al [10] developed Woebot to provide psychotherapy and education focusing on depression and anxiety disorders. Other prior work has also explored the use of CAs to provide educational support. Swartout et al [11] created SimCoach to educate veterans and their families about PTSD and depression. SimCoach aims to provide tailored knowledge based on users’ needs, preferences, and concerns.

While there has been an increasing interest in using CAs for mental health support, there is a lack of understanding regarding how CAs can be designed for sustained user engagement and adherence [39,40]. Specifically, there has not been much work on designing CAs to support the continuous self-management of individuals with PTSD. This is a serious knowledge gap, given PTSD can be a chronic condition. This work aims to address this knowledge gap by designing and evaluating PTSDialogue specifically to support the continuous self-management of individuals living with PTSD.

## Methods

### PTSDialogue

#### Overview

**Figure 1:**
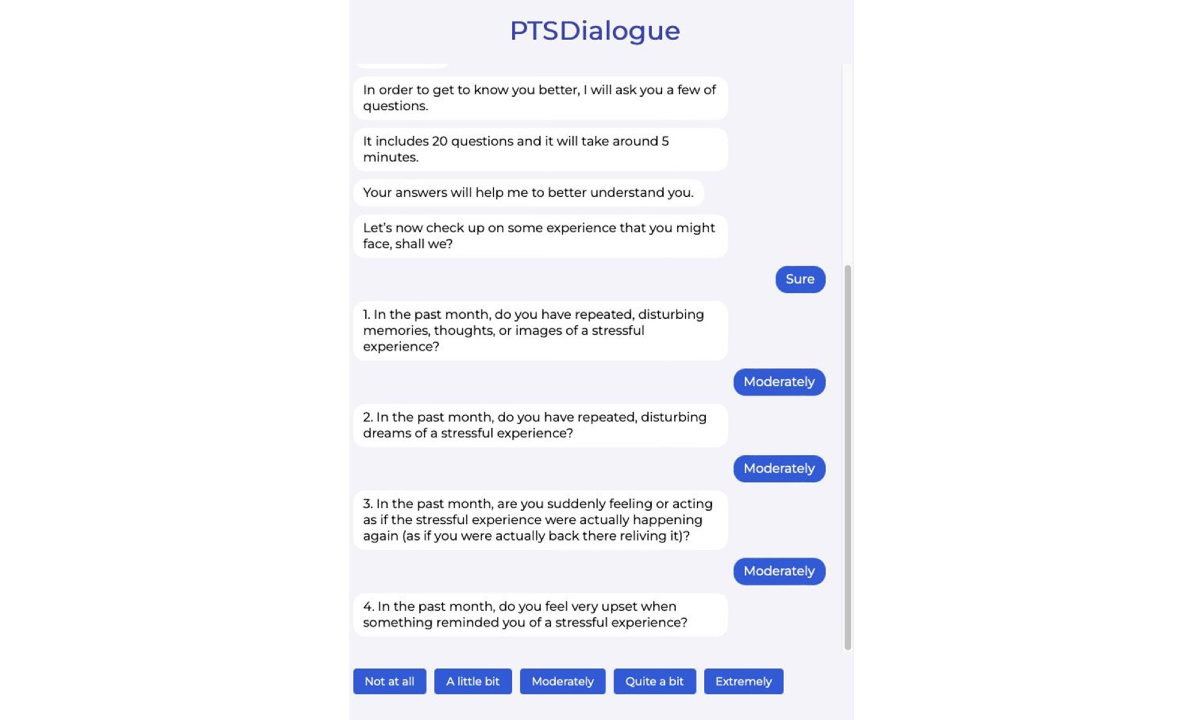
The take assessment module provides a checklist to assess symptom severity. PTSD: posttraumatic stress disorder.

**Figure 2:**
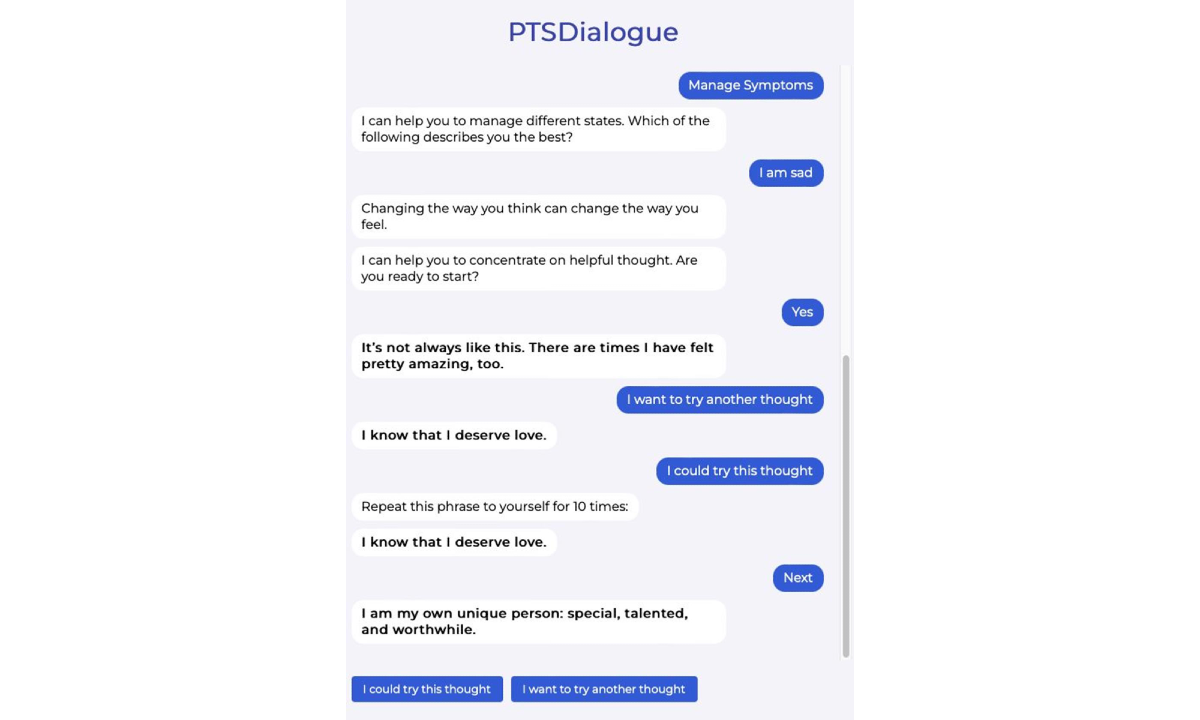
The content in the manage symptoms module provides a number of strategies to manage PTSD symptoms. PTSD: posttraumatic stress disorder.

**Figure 3:**
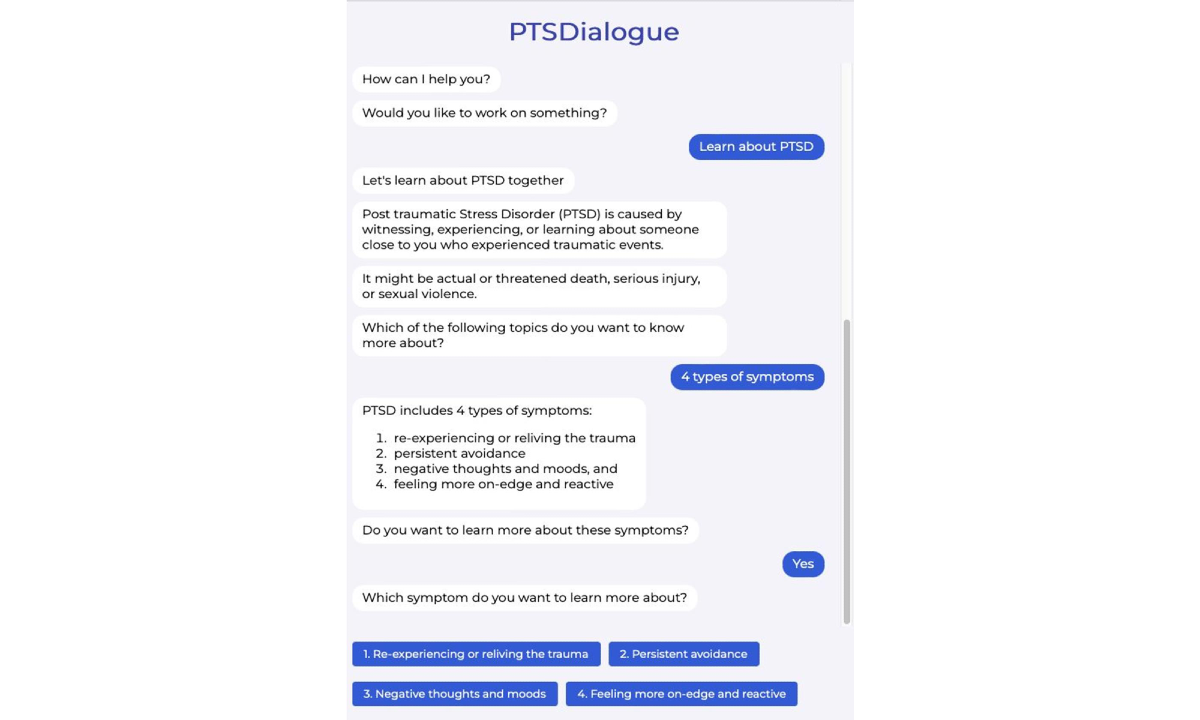
The learn about PTSD module aims to inform users about causes, symptoms, and management strategies for PTSD. PTSD: posttraumatic stress disorder.

**Figure 4:**
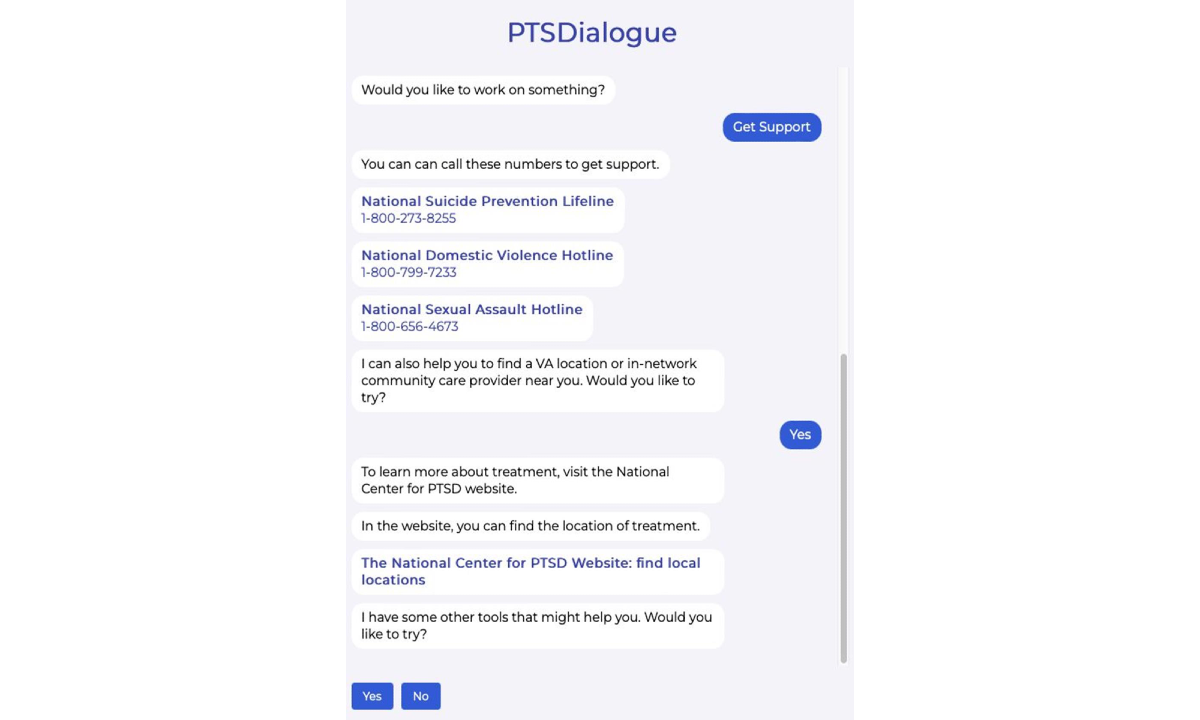
The get support module provides information for a number of resources (eg, phone numbers to helplines). PTSD: posttraumatic stress disorder. VA: veterans affairs.

We have implemented PTSDialogue as a web-based prototype. For implementation, content from PTSD Coach has been adapted [21]. This has resulted in 6 modules in PTSDialogue: take assessment, manage symptoms, get support, learn about PTSD, track progress, and daily symptom checker. Figures 1-4 show examples of different modules in PTSDialogue. The take assessment module provides a self-report measurement based on PTSD Checklist for DSM-5 (PCL-5) [41] for determining symptom severity. The manage symptoms module provides several coping tools. The get support module provides resources, including phone numbers to helplines and Veterans Affairs locations or in-network community care providers. The learn about PTSD module educates users on the causes, symptoms, and management strategies for PTSD. The track progress module shows a history of symptom severity based on self-assessment scores. The daily symptom checker module performs daily self-assessments to check the level of distress.

PTSDialogue is a finite-state CA. Each module is implemented as a set of decision trees. In these decision trees, each node represents a turn-taking point. A node can contain messages and questions. The edges indicate potential branching options based on user inputs. The resultant turn-taking, dialogue-based communication leads to highly engaging interactions with users. Given that each interaction path is predictable in a finite-state CA, it allows for optimizing user experiences while being grounded in evidence-based interventions and minimizing risks.

#### Addressing Ethical and Safety Concerns

It is critical to consider ethical and safety concerns when designing CAs for vulnerable populations. However, current CAs frequently fail to provide a sufficient safety policy [42]. Maintaining safety can be particularly challenging for unsupervised CAs that allow unrestricted user inputs and generate automated responses. PTSDialogue, by design, does not allow users to enter free-form text inputs to mitigate risk, given its limited capabilities to handle emergencies (eg, inputs indicating suicidal ideation). The finite-state CA limits user inputs to predefined options during interactions (eg, the question “Do you want to learn more about these symptoms?” leads to 2 options: “Yes” or “No”). This ensures that each generated response is predictable and appropriate, which is critical given the potential sensitivity of trauma events.

#### Personas

Sustaining long-term engagement with users requires developing an intimate, trustworthy connection with individuals [43]. Perceived social presence is critical in developing strong trust with users [32,33,44,45]. Previous research has shown that using personas and consistent interaction styles can assist in developing a more engaging and favorable connection between users and CAs [32,43,46]. A CA with an empathic persona, in particular, can assist users in efficiently managing their mental health [47]. Likewise, a friendly persona can aid in forming a more favorable connection between users and a CA compared to a neutral persona [43].

**Figure 5:**
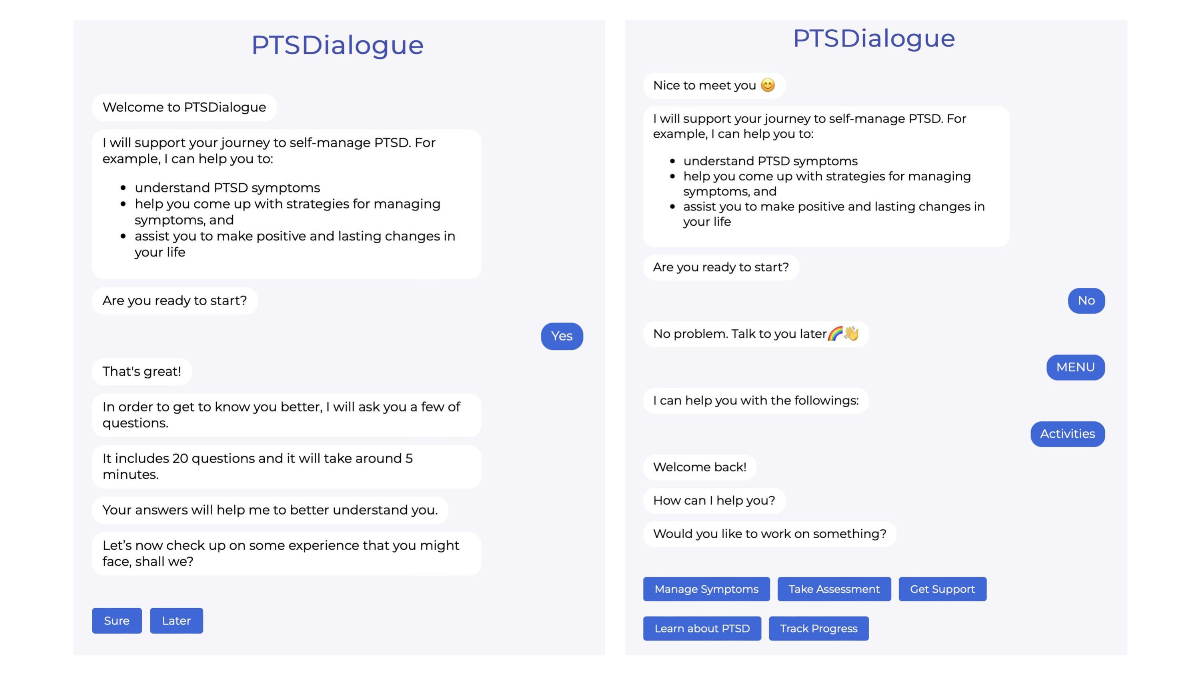
Interactions with both personas in PTSDialogue: the professional persona with a formal and neutral interaction style (left) and the friendly persona with an informal interaction style (right). PTSD: posttraumatic stress disorder.

PTSDialogue has 2 personas to enable different interaction styles and social presence (ie, professional vs friendly). PTSDialogue allows users to choose a persona at the beginning of a session. Figure 5 shows example interactions with both personas. The first persona focuses on being professional, straightforward, and precise, with a formal and neutral communication style. The second persona aspires to be cheerful, friendly, and open, with an informal interaction style with emojis. All messages follow the interaction style of a given persona, but the message content is similar across personas.

### Recruitment

#### Overview

In this study, we focused on collecting expert feedback regarding PTSDialogue, given it focuses on a vulnerable population [15]. To identify potential subjects, Google Scholar was used to identify experts focusing on mental health and technology. Our prior research connections were also used to identify experts. Specifically, we leveraged the research networks of coauthors from the National Center for PTSD to identify potential participants. Recruitment emails were then sent to potential participants providing details about the study. For the study, we recruited 10 clinicians in total. The sample size is consistent with that of prior works [48-50]. All participants have completed their doctoral degrees and have prior experience in PTSD care. Table 1 summarizes participant information.

**Table 1:** Participant information.

ID	Gender	Age (years)	Education	Self-reported level of experience in PTSD^a^ care
P1	Female	35-40	Doctoral degree	Extensive experience
P2	Male	35-40	Doctoral degree	Extensive experience
P3	Female	41-44	Doctoral degree	Extensive experience
P4	Male	31-34	Doctoral degree	Good experience
P5	Female	35-40	Doctoral degree	Extensive experience
P6	Female	35-40	Doctoral degree	Extensive experience
P7	Female	35-40	Doctoral degree	Extensive experience
P8	Female	35-40	Doctoral degree	Extensive experience
P9	Female	25-30	Doctoral degree	Some experience
P10	Female	35-40	Doctoral degree	Good experience

^a^PTSD: posttraumatic stress disorder.

#### Procedures

For the study, our goal was to collect expert evaluation data regarding the acceptance and feasibility of using PTSDialogue to support the self-management of individuals living with PTSD. We conducted remote, one-on-one, semistructured interviews with clinical experts to gather insight into the use of CAs. At the beginning of the interview, demographic and expertise information was collected from the participant using a web-based survey. The web-based PTSDialogue prototype was then shared with the participant so that they could interact with different functionalities and features. We encouraged them to “think aloud” as they interacted with the prototype [51]. Participants also shared their screens throughout the interaction session.

As the participants interacted with the prototype, follow-up questions were asked to clarify their “thinking aloud” comments. A semistructured interview script was also used to gather insights and feedback from the participants. To better contextualize feedback from the participants, we asked questions regarding their experience, expectation, and perceived challenges in providing adequate support for individuals living with PTSD. Their experience and expectations regarding CAs, including if they have used any CA in the past and what challenges they faced in interacting with CAs before, were also discussed. Their opinion and feedback regarding the feasibility and acceptance of PTSDialogue were then explored. Potential suggestions for new features or any recommended changes to improve the usability and acceptance of PTSDialogue were also explored. We asked their thoughts on how they might integrate PTSDialogue with existing clinical workflows and practices for PTSD. Our interview was closed by exploring their feedback and recommendations for technology designers and developers aiming to support vulnerable populations. Each interview was around 60 minutes in duration, and no compensation was provided for participating in this study.

All interviews were audio-recorded and transcribed by the first author. All transcripts were analyzed by a bottom-up thematic analysis using a qualitative interpretivist approach [52]. This data analysis was conducted in 2 stages. During the first stage, 2 coauthors separately reviewed all transcripts and identified keywords within participant responses. In the second stage, the 2 coauthors then iteratively merged identified keywords and categorized them into themes. Any disagreement regarding the generated themes was addressed through follow-up discussions with these coauthors.

### Ethics Approval

The study was approved by the Penn State University institutional review board (STUDY00021763). We used a verbal consent process for the study. Study details and consent documents were shared prior to interview sessions to provide participants adequate time to familiarize with the data collection and analyzing steps.

We took several steps to ensure participant privacy and confidentiality. The study data were deidentified. A randomly generated number was used as an identifier during the data analysis process. To limit data collection scope, we did not collect any video data during the interviews. Only the research team members had access to the collected study data. There were no costs to the participant for their involvement in this study.

## Results

The following section presents major themes from the interview data. These themes include acceptance and feasibility of PTSDialogue as well as identified design requirements for PTSDialogue as a supportive tool.

### Acceptance and Feasibility of PTSDialogue

Most participants (9 out of 10 experts) agreed that PTSDialogue could be used as a supportive tool for individuals living with PTSD. They also identified how features in PTSDialogue can support different self-management needs and strategies for this population. P4 noted that “self-guided coping skills will be super helpful for any population and particularly this population because they avoid so much.”

Participants also noted the usefulness of interactive information as presented in PTSDialogue compared with the traditional resources:

…rather than a handout, this is a little bit more palatable, especially for people in their 20s or 30s, who are more used to texting or are more used to this kind of presentation of information. It just keeps some more engaged with the messages popping up.

P9

P8 appreciated that PTSDialogue is “simplified but not too simple that it loses the value of getting someone to engage in therapy or some kind of professional help.”

P6 emphasized the importance of correctly identifying user intention and needs: “[PTSDialogue is] trying to guess at the user's intention, and it does not always land right.”

While our design process primarily focused on supporting individuals with PTSD, the study participants also noted how the technology could be beneficial for care partners and clinicians. P5 commented that PTSDialogue “might be [useful] for a care partner who wants to learn about PTSD.”

This is particularly important given that care partners play a critical role in the early stages: “care partners are often the ones that are seeking help because the number one symptom of PTSD is avoidance” [P5].

Participants highlighted how PTSDialogue could be integrated into existing clinical workflows and practices. P1 noted that PTSDialogue could be used to support skill acquisition: “it could be helpful in PTSD treatment [...] as homework [...] It is easier than giving a worksheet [to individuals with PTSD].”

Participants also pointed out that given the lack of resources, professional support for individuals living with PTSD is often infrequent and inadequate. PTSDialogue could address this gap by providing support between clinical visits. P2 commented that PTSDialogue can be particularly helpful in this context as it has “a set of skills that they can learn, and that help them at the moment.”

Participants also expressed excitement about the potential usefulness of data collected from PTSDialogue for status monitoring and personalized treatment. P8 commented that “we can track it together during each session. It is very beneficial for the providers as well.”

P10 suggested, “it is helpful for engagement, especially if you [...] send [data] to their provider. That's useful.”

Participants also noted the potential of PTSDialogue to provide support for underserved and difficulty to reach individuals. P7 commented that they “would be interested in disseminating [PTSDialogue] to patients because not everybody wants to engage with us. They will talk to their primary care doctor but do not want to talk to us.”

### Assessment of Features and Interactions Supported by PTSDialogue

#### Interactivity and Tailored Suggestions

The design of PTSDialogue focuses explicitly on supporting interactivity instead of the passive delivery of information. It uses short dialogues that are designed to be conversational in style. Participants noted that the resultant interactions can lead to better usability and sustained engagement. For example, P9 commented that PTSDialogue “was easy to use. It was self-intuitive [...] I thought it was very user-friendly.”

Similarly, P5 pointed out that dialogue-based interaction “broke down different branching options based on what my needs were. So, I felt like a person coming in and paying attention to what I say.”

Furthermore, 3 interview participants noted that dialogue-based interaction could be fun. There were also suggestions to make content gamified to sustain engagement continuously: “the idea of making it a little more interactive and gamifying it” [P4].

These findings indicate the potential advantage of highly interactive CAs in supporting individuals with PTSD over time.

To generate resultant interactions, PTSDialogue uses actionable suggestions. It allows users to choose what they want based on their perception of the information provided. Following a user’s input, PTSDialogue provides tailored activities and intervention strategies for a given session. Four interview participants identified that curated interactivity by actionable suggestions helps to improve user experience. P9 compared the experience of using PTSDialogue with other mobile health apps:

…there is [a] similar app, which also provides mental health help. But the problem is that whenever you say that, you have a symptom [...] same as what you had last time, it shows you the same educational material every time. So I think that is the big reason why people do not go back for a second time because, like, 'Oh, I have read that already. I know what it is going to tell me.' And, I think this one [PTSDialogue] is a little bit different than that. I like it.

To provide properly tailored suggestions, future version of PTSDialogue should consider a user's prior activities and preferences.

However, collecting such data through a CA can lead to serious privacy concerns. P1 noted that people living with PTSD “are so concerned about privacy, and [collecting trauma and symptom data] might feel sort of intrusive.”

It is particularly challenging given the sensitivity of traumatic events. P9 commented that “even in in-person therapy, people are usually not comfortable with talking about their trauma a lot of times.”

P9 also speculated that “maybe it will be different [with PTSDialogue compared with in-person sessions] because they think that they are typing into a space where no one can see.”

#### Identifying User Needs and Intentions

PTSD symptoms manifest differently for different individuals. To provide effective support, it is important for a CA to correctly identify user needs and intentions at a given time. Two interview participants cautioned against misidentification of users’ intentions, which might lead to inappropriate responses from the CA. P3 commented that “it's actually kind of activating my nervous system in uncomfortable ways. Because the assumptions are being made about what my thoughts actually are, and what thoughts I mean would be helpful.”

A CA should deliberate on users’ needs and intentions to prevent making hasty assumptions.

Given the nuances of PTSD, a CA for individuals living with PTSD should focus on interaction steps that can unambiguously identify users’ intentions and needs for each given usage session. Furthermore, regular symptom assessment is important to identify a user’s overall well-being trajectory as well as provide personalized interventions. P7 pointed out that it is essential to:

…give an overview of different kinds of traumatic events that people can have. The person is actually asked to select the most stressful experience. And when they answer the questions, they have got that particular stressful event in mind. Because if you do not do that, you will probably get a very high false-positive rate. To reduce your false positive rate, you want to anchor with a specific traumatic event and use either the life events checklist or the criteria that go along with PCL-5.

From this testimony, it is clear that a CA in this context should have well-designed interactions that can meaningfully collect such assessments from users.

Participants further highlighted the need for transparency on how trauma and symptom data will be used to support the user. P1 noted that users will be reluctant to share data if the perceived benefit is not high “if the content I am getting back based on disclosing that information is not all that different*.*” P9 also suggested limiting data collection to minimize risks and improve user trust in CAs: “I am wondering what [PTSDialogue] could do with [information regarding specific trauma experience] other than increase liability for a privacy breach.” CAs should, thus, be transparent in conveying how the information will be used to support user needs as well as how the information will be stored securely.

#### Personas

PTSDialogue has 2 kinds of personas: professional and friendly. The professional persona represents professionals using formal language. The friendly persona represents friends using informal language, including emojis. The majority of interview participants (8 out of 10 experts) emphasize the importance of persona. Seven interview participants prefer the friendly persona. P8 describes the friendly persona as “more relatable and more supportive [...] almost feels like you are talking to someone.”

However, 2 interview participants had concerns with the friendly persona being casual. P10 thought that emojis in the friendly persona might “make [people living with PTSD] feel like the CA is less professional or less accurate. They have some stigma, associating a lot of emojis with social media.”

P7 noticed that certain emojis have different meanings depending on users’ backgrounds. For example, the pray emoji, which looks like 2 hands placed firmly together, might cause misunderstanding: “I could see people thinking of that as like a prayer kind of religious thing.”

P8 also pointed out:

…there are people that will like the professional persona because there are people that struggle with feeling like other people who are too supportive, or they have trust issues. So, they want the facts. They do not want feelings attached to it. However, with the friendly persona, some of the emotion of `I am here to support you' is coming out. So they can fully understand what their options are before they engage in it. I think just using the words professional and friendly will not be enough. I think that will be confusing.

At the same time, P5 suggested humor as an important communication technique:

…the reason I am [suggesting to] offer some levity is because PTSD is such a sobering condition. It is terrible. It is literally the worst thing that people have happened in their life and now [it] terrorizes them indefinitely. But finding a way to respectfully implement humor and creativity is usually appreciated. Emojis are more supportive and keep the product that will keep people engaged.

Overall, it is crucial for PTSDialogue to support multiple personas with different interaction styles as well as correctly identify which personas would better fit the need of a given user.

#### Critical Role of Empathy

There was consistent feedback from our participants regarding the importance of empathy in a CA aiming to support individuals with PTSD. While we focused on including empathetic responses in PTSDialogue during the design phase, 4 participants found them inadequate. P5 commented, “I would have liked to see a little bit more empathy built into the responses.”

Two participants wanted empathy and support in PTSDialogue that will mimic human-to-human interactions. P2 commented, “I'm not really noticing the illusion of talking to somebody [...] I don't get the sense that there's genuine care.”

Participants also provided suggestions for integrating empathy better and humanizing PTSDialogue interactions. P1 advised that even the friendly persona should “lighten up or make it more conversational in tone.”

Two participants suggested using interactions from therapy sessions to better model empathetic responses from PTSDialogue. P9 recommended “to emulate real-life therapy adding empathic statements [and] simulate more of a real-life when you are talking to somebody.”

#### Balancing Educational and Skill Acquisition Goals

Recent mobile health work has suggested prioritizing “doing” over providing knowledge [53]. Such interactions will focus on practicing skills instead of providing background knowledge or explanation. Mohr et al [53] argued that such a “learning-by-doing” approach is better suited for brief and frequent user engagement, which can lead to more effective skill acquisition. As such, there is a need to balance the educational and skill acquisition goals in a CA aiming to provide therapeutic support. PTSDialogue follows the “learning-by-doing” approach with interactions that actively engage users in practicing skills (eg, thought shifting). Users are not required to go through an explanation or background knowledge before engaging with these skills.

However, 4 participants wanted PTSDialogue to focus more on the background and educational support. P2 commented, “there [is a] need to [provide] more background information about what these skills are. These seemed a little bit too kind of stripped-down.”

P2 also suggested that PTSDialogue should “explain to users how they are supposed to use [these tools] [...] Otherwise, it is just going to feel open-ended.”

Based on these findings, it will be important to design interactions that can unobtrusively provide useful background information in PTSDialogue.

#### Risk Management

Given that the target audience for PTSDialogue is a vulnerable population, effective risk management is a critical design criterion. PTSDialogue provides a “get support” feature that points to useful resources including phone numbers to the Suicide Prevention Lifeline. However, 2 participants suggested having resources that are more situation-appropriate for individuals in need. P7 commented:

…[crisis numbers] would throw me off a little bit. Because if I was a patient, this makes it sound like, especially when I first see the Suicide Prevention Lifeline, I may, or even if I have really serious PTSD, I may or may not be having suicidal thoughts [...] It feels a little bit like overkill because these may not be appropriate resources at all.

There was also concern about users potentially reliving trauma during the data collection process. While such data would lead to better tailored interventions from the CA, recalling specific events might be challenging for some users. P10 commented:

…anchoring trauma experience is a tremendous responsibility. There is a lot of damage that can be done with inappropriate or incorrect responses to patients with PTSD. Some patients with PTSD need certain kinds of responses, and others need other kinds depending on severity. So, it is very hard. I think this task is difficult. [CAs] have to be sophisticated because there is a high risk for harm.

However, some participants thought that asking about traumatic experiences might not lead to increased risk. P8 pointed out:

…the new research now tells us it does not matter if the person talks about the account or not, you will get the same result. So that, the standard right now is you can do trauma-focused treatment without having to talk about your trauma. So should [a CA ask about traumatic events]? It would not make a difference.

To minimize user risk, the current version of PTSDialogue does not collect data about specific traumatic events.

## Discussion

### Design Implications

#### Overview

Through this work, we established the feasibility and acceptance of using PTSDialogue for this specific population of users. To this end, interview data were collected from 10 clinical experts. Experts agreed that PTSDialogue can be used to support self-management of individuals living with PTSD. We also identified key features and interactions supported by PTSDialogue that contribute to supporting self-management for our target population. Based on these findings, design recommendations were provided for future CAs aiming to support self-management of vulnerable populations.

#### Dynamic and Tailored Content

To sustain user engagement, it is important for a CA to provide dynamic and tailored content to users. P9 mentioned that eHealth technologies often tend to provide static material to users, which can lead to user attrition and low adherence. Dynamic content is particularly important for a CA to support brief but frequent interactions with users. We suggest that CA designers should come up with different interactions and content based on identified intervention strategies, which then can be delivered based on predefined conditions (eg, symptom type and severity).

It is also important to provide tailored interactions that match users’ needs and intentions. P2 noted that users get discouraged when CAs provided invalid or nontailored interactions. It is particularly important to remember prior user decisions and preferences. For example, suggesting strategies previously chosen by the user for a given condition can lead to efficient interactions*.* As such, CA designers should consider that responses from a CA are contingent upon the user’s input. The resultant perceived message contingency can lead to better user engagement [54].

#### Assessment and Monitoring Stability

Assessment and stability monitoring are key features for a CA aiming to support vulnerable individuals with chronic conditions. P8 mentioned that people living with PTSD often want to know about how they are doing over time, even when they are not doing so well. Clear communications of wellness trajectory can also motivate users toward effective self-management. Furthermore, assessment data are critical for effective support from a CA. For example, a CA can provide specific intervention strategies based on symptom severity over time (eg, a high score in anger over 3 days). Keeping track of resources used by the user and subsequent changes in stability will also allow the CA to provide more effective tailored interventions for similar symptoms in the future.

Furthermore, assessment and stability monitoring in the CA can lead to better integration with existing clinical workflows. By collecting regular symptom data, a CA can help to make better informed clinical decisions. As such, CA designers should identify condition-specific interactions that can lead to frequent assessment as well as communicating key metrics and trajectories to users. Furthermore, it is critically important that care is taken to ensure that such assessment behaviors do not contribute to emotional harm, such as reliving trauma. While the current prototype of PTSDialogue does not collect information about specific traumatic events, future work should investigate how technological systems can effectively balance data collection while protecting users’ emotional safety.

#### Expectation Management

It is important to clearly convey the abilities and limitations of a CA to users. Otherwise, users might overestimate the capabilities of CAs. This can lead to unrealistic user expectations of human-level support and communication capabilities from a given CA. In this case, any error from CAs will be judged harshly by the user, which can have a significant negative impact on perceived usability and user engagement. Furthermore, overestimation of capability can be a serious issue for a CA aiming to provide support for vulnerable populations. While designing CAs for these populations, it is critical to ensure that users understand the interactions in CAs are not provided by a human, and as such, support from CAs is limited, specifically when it comes to emergencies. Designers can help to manage user expectations by clearly conveying how the CA makes certain decisions. Explainability can lead to a better understanding of CA capabilities and limitations.

#### Addressing Privacy and Trust Concerns

It is critical to address privacy and trust concerns for a CA aiming to support this population. Prior work has found CAs can lead to more forthcoming self-disclosure from individuals [35]. However, identifying privacy concerns with different types of information will be critical for CAs to ensure the balance between data collection and tailoring support. Future work should focus on identifying what information individuals will be comfortable sharing with CAs. To reduce the risk of harm, researchers and developers should engage with clinical experts early in the CA design process. It is also important to follow established therapeutic resources and guidelines to generate content for CA interactions. During the interaction design process, it is advisable to collaborate with clinical experts to refine content and dialogue when delivering materials that address sensitive topics. Following the implementation of the CA, it is critical to seek feedback from clinical experts before deployment to vulnerable individuals.

### Limitations

The study has a number of limitations, which should be kept in mind while interpreting the findings and outcomes presented in the paper. First, we collected interview data from a small number of clinical experts. While the number of participants in our study is consistent with prior work [48,49,55,56], future work should explore data from a larger sample of experts using other methods (eg, surveys). Additionally, our data collection was limited to clinical experts. While this approach provides useful insights from experts’ perspectives, it is critically important for future studies to collect interaction and acceptance data from individuals living with PTSD as well.

### Conclusions

In this paper, we present an expert evaluation of PTSDialogue—a finite-state CA to support the self-management of individuals living with PTSD. Semistructured interview data were collected from 10 clinical experts following their interactions with PTSDialogue. Our findings show that experts agreed that PTSDialogue can be used to support the self-management of individuals living with PTSD. The use and efficacy of different features and interactions supported in PTSDialogue were further identified and assessed. Based on these findings, we have also provided design recommendations for CAs aiming to support vulnerable populations. We believe that well-designed CAs can reshape effective intervention delivery and consequently help to address the current treatment gap in mental health.

## Abbreviations

CA: conversational agent
PCL-5: PTSD Checklist for DSM-5
PTSD: posttraumatic stress disorder
SA: supportive accountability

## Data Availability

The data set is not publicly available due to participant privacy and confidentiality concerns. However, a subset of the generated data is available from the corresponding author upon reasonable request.
